# Influence of bacterial components on the developmental programming of enteric neurons

**DOI:** 10.14814/phy2.14611

**Published:** 2020-11-13

**Authors:** Jelena Popov, Julia Bandura, Filip Markovic, Rajka Borojevic, Varun C. Anipindi, Nikhil Pai, Elyanne M. Ratcliffe

**Affiliations:** ^1^ Division of Gastroenterology and Nutrition, Department of Pediatrics McMaster University Hamilton ON Canada; ^2^ McMaster Integrative Neuroscience Discovery and Study Graduate Program McMaster University Hamilton ON Canada; ^3^ Department of Physiology University of Toronto Toronto ON Canada; ^4^ Farncombe Family Digestive Health Research Institute McMaster University Hamilton ON Canada; ^5^ Department of Medical Sciences McMaster University Hamilton ON Canada

**Keywords:** bacterial components, development, enteric nervous system, enteric neural crest‐derived cells

## Abstract

**Background:**

Intestinal bacteria have been increasingly shown to be involved in early postnatal development. Previous work has shown that intestinal bacteria are necessary for the structural development and intrinsic function of the enteric nervous system in early postnatal life. Furthermore, colonization with a limited number of bacteria appears to be sufficient for the formation of a normal enteric nervous system. We tested the hypothesis that common bacterial components could influence the programming of developing enteric neurons.

**Methods:**

The developmental programming of enteric neurons was studied by isolating enteric neural crest‐derived cells from the fetal gut of C57Bl/6 mice at embryonic day 15.5. After the establishment of the cell line, cultured enteric neuronal precursors were exposed to increasing concentrations of a panel of bacterial components including lipopolysaccharide, flagellin, and components of peptidoglycan.

**Key Result:**

Exposure to bacterial components consistently affected proportions of enteric neuronal precursors that developed into nitrergic neurons. Furthermore, flagellin and D‐gamma‐Glu‐mDAP were found to promote the development of serotonergic neurons. Proportions of dopaminergic neurons remained unchanged. Proliferation of neuronal precursor cells was significantly increased upon exposure to lipopolysaccharide and flagellin, while no significant changes were observed in the proportion of apoptotic neuronal precursors compared to baseline with exposure to any bacterial component.

**Conclusions and Interfaces:**

These findings suggest that bacterial components may influence the development of enteric neurons.

## Graphical abstract

Exposure to flagellin and D‐gamma‐Glu‐mDAP affected the proportion of enteric neuronal precursors that developed into serotonergic neurons. Nitrergic neuron development was influenced by exposure to lipopolysaccharide, flagellin, muramyl dipeptide, and D‐gamma‐Glu‐mDAP, while proportions of dopaminergic neurons remained unchanged. Proliferation of neuronal precursor cells was influenced by lipopolysaccharide and flagellin, while apotosis remained unaffected by exposure to each of the bacterial components.

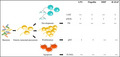

## 1 INTRODUCTION

The enteric nervous system (ENS) plays a critical role in maintaining gut homeostasis and mediating interactions between the contents of the intestinal lumen and the internal environment of cells and tissues. The ENS participates in the regulation of blood flow (Neild et al., 1990), initiation of appropriate responses to sensory stimuli (Burns et al., 2016), crosstalk with the immune system (Hao & Young, 2009), and controlling gut motility (Hao & Young, 2009). Normal development of the ENS in early life is, therefore, critical for the postnatal function of the gastrointestinal tract.

As a result of its highly integrative and complex role, ENS development involves the tight regulation of precursor cell differentiation, neurite growth, and establishment of appropriate neural networks. Colonization of the gut is mediated by two populations of enteric neural crest‐derived cells (ENCDCs) with unique patterns of migration (Burns and Le Douarin, 1998; Hao et al., 2009; Le Douarin & Teillet, 1973). Coordinated migration is essential for appropriate innervation throughout the gastrointestinal tract, with defects in these pathways giving rise to regions of aganglionosis and pathological manifestations (Young et al., 2006).

The majority of ENS precursors originate from vagal ENCDCs which invade the foregut mesenchyme at embryonic day (E) 9.5 in mouse development and migrate rostra‐caudally (Burns & Le Douarin, 1998; Kapur et al., 1992; Le Douarin & Teillet, 1973; Miyahara et al., 2011; Yntema & Hammond, 1954). In contrast, sacral ENCDCs display delayed entry into the gut, undergo caudo‐rostral migration, and innervate mostly the hindgut (Burns & Le Douarin, 1998; Le Douarin & Teillet, 1973; Miyahara et al., 2011). Linear migration of precursors is complete by E14 (McKeown et al., 2001), followed by inward migration toward the mucosa and differentiation (Jiang et al., 2003), ultimately giving rise to the myenteric plexus and subsequently, the submucosal plexus (McKeown et al., 2001). It is around this time, at E15.5, that ENCDCs are most abundant (Binder et al., 2015).

Enteric neurons of different phenotypes are born at different times of development (Pham et al., 1991). ENCDCs that colonize the stomach and proximal small intestines are the first to develop into phenotypes that define the mature ENS, and begin to arise as the migrating wavefront of ENCDCs continues to invade the caudal most regions of the gut (Conner et al., 2003; Hao & Young, 2009; Young et al., 1999). Serotonergic, or 5‐hydroxytryptamine (5‐HT), neurons are considered early‐born and their appearance coincides with the first wave of ENCDC invasion of the foregut (Bergner et al., 2014; Pham et al., 1991). Dopaminergic (TH) and nitrergic (nNOS) neurons both have dual populations of earlier and later‐born neurons, with ongoing development into the postnatal period (Bergner et al., 2014; Branchek & Gershon, 1989; Hao et al., 2010; Li et al., 2004; Pham et al., 1991; Sang & Young, 1996).

This critical window of proliferation and phenotypic development of enteric neural precursors in the perinatal period coincides with microbial colonization of the gut. Previous work has shown that enteric neurons of germ‐free (GF) mice exhibit structural (Collins et al., 2014) and electrophysiological (McVey Neufeld et al., 2015) abnormalities which may be reversed upon conventionalization with specific pathogen‐free (SPF) microbiota. Particularly intriguing are findings that demonstrate these abnormalities arise as early as postnatal day 3 in GF mice compared to altered Schaedler flora (ASF) mice (Collins et al., 2014). Further work has found that intestinal microbiota is not limited in its effects on influencing only the neurons of the ENS; instead, it also serves as an essential component for the normal development of the mucosal glial cell network (Kabouridis et al., 2015). It has been shown that GF mice exhibit a significant reduction in the number and density of enteric glial cell connections and that conventionalization after the perinatal period normalizes the glial cell networks (Kabouridis et al., 2015). These early life disruptions in ENS physiology support the concept that input from even a simple intestinal flora is sufficient for influencing ENS development.

While research to date suggests that intestinal microbiota can play a role in the normal development of the ENS, the mechanisms by which this occurs have not yet been elucidated. The current study tested the hypothesis that bacterial components interact directly with enteric neuronal precursors, and directly mediate the developmental programming of enteric neurons. We report that exposure of neuronal precursors to a panel of common bacterial components appears to influence developmental programming through direct interactions in vitro. Specifically, bacterial components appear to promote development into serotonergic and nitrergic, but not dopaminergic, phenotypes. Bacterial components stimulate proliferation under certain conditions; however, apoptosis remains unaffected. These findings suggest that bacterial components can influence the development of enteric neurons.

## 2 METHODS

### 2.1 Animals

Timed pregnant SPF C57Bl/6NTac mice were ordered from Taconic Biosciences, Inc. (Rensselaer, NY, USA), and maintained on ventilated racks in ultraclean units in the McMaster Central Animal Facility. The day of plug detection was considered E1. Timed pregnant dams (*n* = 6) were anesthetized using isoflurane, sacrificed by cervical dislocation, and male and female fetuses were dissected at E15.5. Treatment of animals and all experiments were conducted in accordance with the McMaster Animal Research Ethics Board (AUP: 140621).

### 2.2 ENCDC Isolation

Timed pregnant SPF dams (*n* = 6) were sacrificed at E15.5, and fetuses (*n* = 48) were extracted under sterile conditions in a biological safety cabinet. Entire fetal guts were isolated and digested with collagenase (1 mg/ml, Sigma C0130) and manual trituration. Cells were passed through a cell strainer to ensure single‐cell preparation. Single cells were incubated with primary antibody rabbit anti‐mouse p75^NTR+^ for 1 hr (1:50, Alomone ANT‐007), and secondary antibody anti‐rabbit IgG microbeads for 15 min (150 µl per 10^7^ cells; MACS Miltenyi Biotec 130‐048‐602). Cells were passed through a magnetic cell separation column (Miltenyi Biotec 130‐042‐202). Negative cells were discarded. Positive cells were plated on 2% fibronectin‐coated plates at a density of approximately 275,000 cells mL^‐1^ in 6‐well plates. Cells were incubated at 37°C, 5% CO_2_. Neurosphere media (NSM+) was supplemented with N2 (1%; Invitrogen 17502048), B27 (2%; Invitrogen 17504044), EGF (20 ng/ml; Invitrogen PMG8041), bFGF (20 ng/ml; Sigma F0291), heparin (0.5 U mL^‐1^; Sigma H3149), and antibiotics streptomycin/penicillin (1%; Invitrogen 15140122) in DMEM/F12 media (Sigma D8437). NSM + media was supplemented with 10% horse serum for the first 24 hr after dissection to facilitate adherence. After 24 hr, the media was changed to standard NSM + media with nutrients and growth factors only. Old NSM + media was replaced with fresh media every 3–4 days. Adherent cells were passaged every 14 days.

### 2.3 Flow cytometry

Cultured cells were stained with p75^NTR+^‐FITC (Alomone Labs ANT‐007F) and 7‐Aminoactinomycin D (7‐AAD; Invitrogen 00–6993–50). All samples were processed using the BD FACSCanto flow cytometer (San Jose, CA, USA) connected to a Hewlett‐Packard computer (Hewlett‐Packard Inc., Palo Alto, CA, USA). Data acquisition and analysis were performed using FACSDiva (BDFACSDiva, Aukland, New Zealand) and FlowJo Software (FlowJo LLC, Ashland, OR, USA).

### 2.4 Preparation of cultured cells for immunocytochemistry

Cultured cells were grown in chamber slides at a density of 275,000 cells mL^‐1^ of NSM + media. Cells were fixed in 4% paraformaldehyde (freshly prepared from paraformaldehyde, pH 7.4), and permeabilized by incubating in blocking buffer (1% PBS, 4% normal horse serum, 0.5% Triton^TM^ X‐100). Cells were exposed to primary antibody overnight at room temperature. Subsequent treatment with secondary antibodies for 3 hr at room temperature in the dark allowed for the visualization of targetted cells.

Primary antibodies included p75^NTR+^ (1:50, Alomone Labs ANT‐007), HuC/D (1:50, Invitrogen A21272), 5‐HT (1:200, ImmunoStar 20080), nNOS (1:500, Cedarlane), TH (1:500, Millipore Sigma AB1542), and phospho‐histone H3 (pH3; 1:500, BioLabs 9705S). Sites of antibody binding were detected by incubation with the following secondary antibodies: donkey anti‐rabbit Alexa Fluor 594 (1:200, Molecular Probes A21207), streptavidin 488 (1:200, Molecular Probes S11223) or streptavidin 594 (1:200, Molecular Probes S32356), donkey anti‐sheep Alexa Fluor 488 (1:200, Invitrogen A11015) or donkey anti‐sheep Alexa Fluor 594 (1:200, Invitrogen A11016), and donkey anti‐goat Alexa Fluor 594 (1:200, Invitrogen A11058). Total positive cells were manually counted and compared to total HuC/D positive cells. Bisbenzimide was used as a nuclear counterstain. The selection of antibodies and immunostaining protocols was based on previous studies (Collins et al., 2014; Li et al., 2011). In control chambers, no immunostaining was seen when primary antibodies were omitted (data not shown).

### 2.5 Detection and quantification of apoptotic cells

Apoptosis was detected using terminal deoxynucleotidyl transferase (TdT)‐mediated 2'‐deoxyuridine 5'‐triphosphate (dUTP) nick end labeling kit (TUNEL; Promega DeadEnd^TM^ Fluorometric TUNEL System G3250, used according to manufacturer's instructions). This assay involves TdT binding to blunt ends of DNA fragments and catalyzing the incorporation of fluorescently labeled nucleotide, fluorescein‐12‐dUTP. Total apoptotic cells were manually counted and compared to total HuC/D positive cells. Bisbenzimide was used as a nuclear counterstain. The proportion of apoptotic cells after exposure to bacterial components were compared to NSM + vehicle control. Treatment with DNase (Promega M6101) was performed for the positive control, and the reverse TdT enzyme was omitted for the negative control.

### 2.6 Exposure to bacterial components

Bacterial components were chosen to represent a range of common intestinal microbiota: lipopolysaccharide (LPS) is a component found in gram‐negative bacteria, flagellin can be found in both gram‐negative and gram‐positive bacteria and the peptidoglycan derivatives muramyl dipeptide (MDP) and D‐gamma‐Glu—mDap (iE‐DAP) are found primarily in gram‐positive bacteria.

Cultures were incubated with LPS (0.001, 0.01, 0.1, 1 µg/ml, Sigma L2630, isolated from Escherichia coli serotype O111:B4), flagellin (0.001, 0.01, 0.1, 1 µg/ml, Enzo Life Sciences ALX‐522–058‐C010, isolated from Salmonella typhimurium strain 14028), MDP (0.01, 0.1, 1, 10 µg/ml, InvivoGen tlrl‐mdp, chemically synthesized), iE‐DAP (0.1, 1, 10, 100 µg/ml, InvivoGen tlrl‐mdp, chemically synthesized), and NSM + vehicle control. Varying concentrations of bacterial components were used to assess concentration‐dependent effects. Bacterial component solutions were prepared in fresh NSM + media. Cells were incubated with the bacterial components for 24 hr; incubation time was determined based on previous studies with LPS and evidence that longer exposure to LPS can increase toxicity (Anitha et al., 2012, 2016; Arciszewski et al., 2008; Burgueño et al., 2016; Coquenlorge et al., 2014). Following incubation with bacterial components or vehicle control, the cells underwent fixation as described above and processing for proliferation using pH3, apoptosis using TUNEL, and chemical coding including 5‐HT, nNOS, and TH. Total positive cells were manually counted and compared to total HuC/D positive cells. Bisbenzimide was used as a nuclear counterstain. The proportion of apoptotic cells after exposure to bacterial components were compared to NSM + vehicle control. Each experimental condition was performed in duplicate (except the TUNEL staining after exposure to flagellin due to technical limitations).

### 2.7 Image analysis

Quantification of enteric neuronal precursors was performed by photographing five regions per slide at 20x magnification using a Leica DMRXA2 microscope with fluorescence (Leica Microsystems Inc, Concord, ON, Canada). Cells were manually counted using Volocity software (Improvision Inc., Montreal, QC, Canada) on a Macintosh computer (Apple Computers, Markham, ON, Canada). All images were coded to ensure investigator blinding of experimental conditions during image analysis.

### 2.8 Statistical analyses

Data are presented as mean ± *SEM*. Statistical analyses were performed using a Mann‐Whitney U with comparisons between culture characteristics of subculture 4 and 5. Data analyses of the effects of varying concentrations of bacterial components on culture characteristics were performed using Kruskal Wallis with Dunn's post hoc test. Significant outliers were removed. The level of statistical significance was set at *p* ≤ .05. All analyses were performed using GraphPad Prism Version 4 for Mac OS X (GraphPad Software Inc., La Jolla California, USA).

## 3 RESULTS

### 3.1 Assessment of ENCDC culture purity and viability using flow cytometry

**Figure 1:**
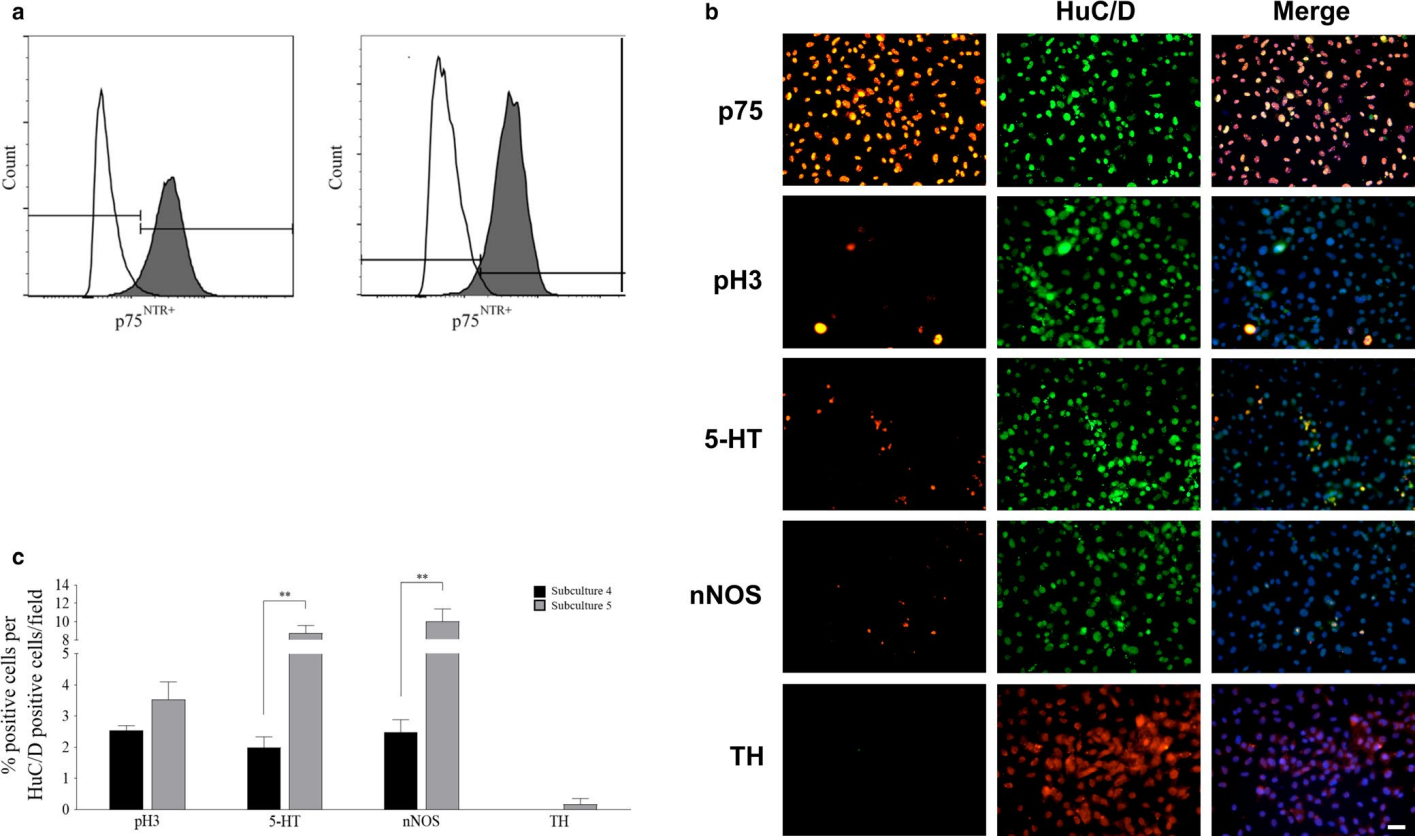
Characterization of ENCDC cultures. Flow cytometry of ENCDC cultures stained with antibodies against the immature neuronal marker, p75^NTR+^‐FITC demonstrated 95.1% p75^NTR+^‐positive, 97.3% viability in subculture 3, and 96.1% p75^NTR+^‐positive, 98.2% viability in subculture 4 (a). Proportion of ENCDC cultures characterized for expression of p75NTR+, pH3, 5‐HT, nNOS, and TH. Scale bar = 20 µm (b). There was a significant increase in the proportion of serotonergic neurons and nitrergic neurons in subculture 5 (gray) compared to subculture 4 (black), but no significant difference in proliferating cells or dopaminergic neurons across the cultures. *p ≤ .05. Values are presented as mean ± *SEM* (c)

The duodenum, jejunum, ileum, and colon segments were harvested from fetuses at E15.5, dissociated into single cells, and immunolabeled using anti‐p75^NTR^ as a marker for enteric neuron precursors (Binder et al., 2015). Cells were grown in culture for three subcultures to generate sufficient enteric neuronal precursors and allow development similar to a perinatal timepoint (Bergner et al., 2014). Flow cytometry analyses were conducted on subcultures 3 and 4 to determine culture purity (Figure 1a) and culture viability using 7‐AAD viability dye (data not shown). Analyses revealed a 95.1% p75^NTR+^‐positive population (Figure 1a) with a 97.3% viability (subculture 3). Similar findings were found when analysis was conducted on the subsequent culture (subculture 4), demonstrating 96.1% p75^NTR+^‐positive cells (Figure 1a) and a 98.2% viability.

### 3.2 Characterization of ENCDC cultures using immunocytochemistry

Cultures were processed for immunocytochemistry to further identify proportions of p75^NTR+^ cells, serving as a marker of enteric neuron precursors (Binder et al., 2015) (Figure 1b). The cultures were also characterized at this baseline timepoint by assessing the extent of differentiation and proliferation (Crone et al., 2003) (Figure 1c). All conditions were co‐stained with pan‐neuronal marker HuC/D as a control for neuronal differentiation, and bisbenzimide nuclear counterstain. Cells co‐expressing p75^NTR+^ and HuC/D composed the largest population (99.3% ± 1.18%), followed by nitrergic neurons (5.83% ± 1.77%), and serotonergic neurons (1.60% ± 0.850%). No dopaminergic neurons were detected. Proliferating precursor cells were present at 4.46% ± 1.56%.

### 3.3 Characterization of enteric neuronal precursor cells in culture

Enteric neuronal precursor cells were further subcultured to obtain a mixed cell population of precursor, early‐born and later‐born enteric neuronal populations. The cultures were then processed for immunocytochemistry in order to identify phenotypes that would be expected at the perinatal timepoint. Two subsequent subcultures (4 and 5) were characterized in order to develop a timeline. Comparison of culture characteristics across subsequent cultures demonstrated a significant increase in the proportion of serotonergic neurons (subculture 4, 2.00% ± 0.780% vs. subculture 5, 8.76% ± 1.86%; *p* = .0079) and nitrergic neurons (subculture 4, 2.49% ± 0.890% vs. subculture 5, 10.0% ± 2.96%; *p* = .0079). No significant differences were found in the proportion of dopaminergic neurons (subculture 4, 0.00% ± 0.00% vs. subculture 5, 0.180% ± 0.410%; *p *> .05) or proliferation of precursor cells (subculture 4, 2.53% ± 0.360% vs. subculture 5, 3.52% ± 1.29%; *p *> .05) (Figure 1c).

### 3.4 Ability of Bacterial Components to Influence Enteric Neuronal Development

**Figure 2:**
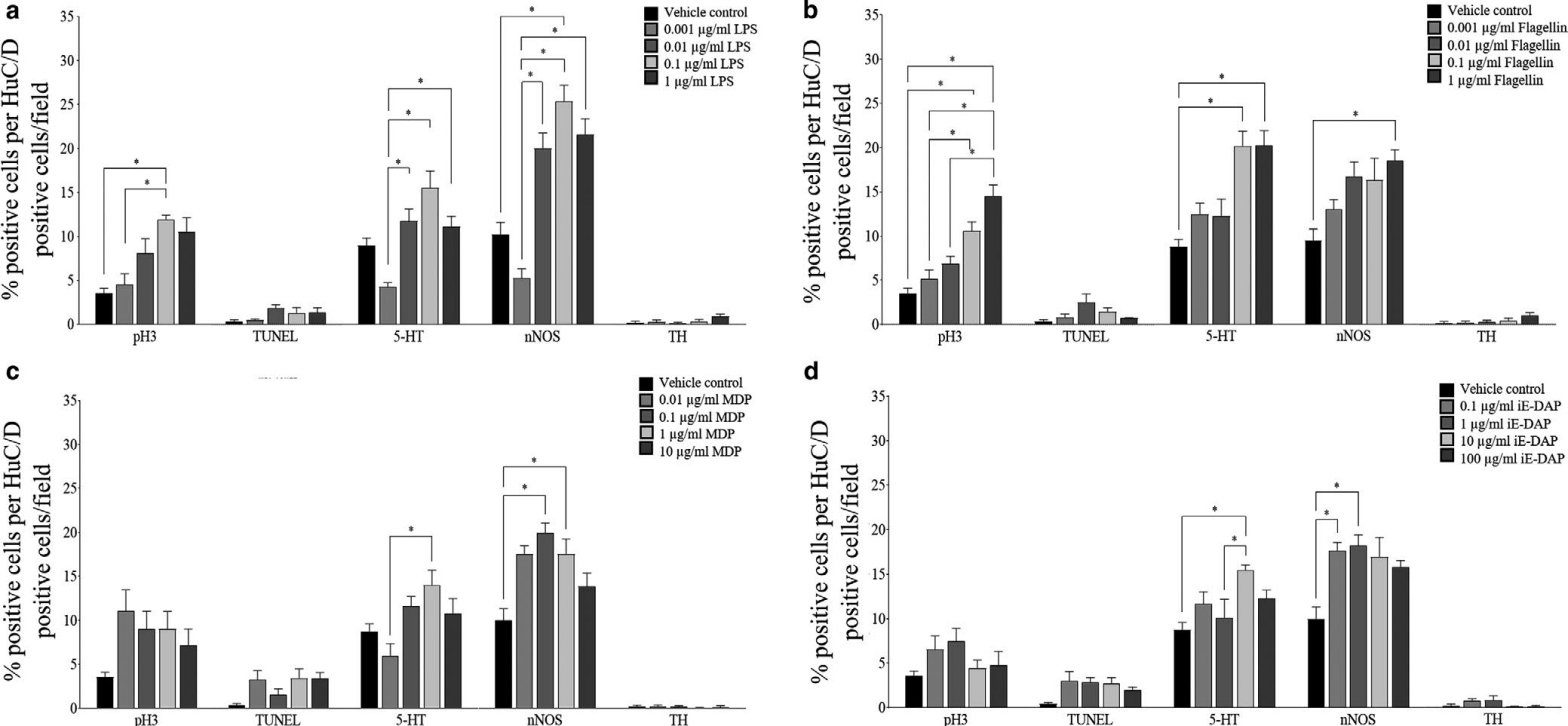
Role of LPS, flagellin, MDP, and iE‐DAP on ENCDC programming. LPS had a significant effect on increasing enteric neuronal precursor proliferation and development into nitrergic neurons, but no significant effect on apoptosis or development into dopaminergic neurons. LPS exhibited a significant effect on increasing serotonergic neurons although this was not significant with the control (a). Flagellin had a significant effect on increasing enteric neuronal precursor proliferation and development into serotonergic and nitrergic neurons. Proliferation appeared to be concentration‐dependent. Flagellin had no significant effect on apoptosis or development into dopaminergic neurons (b). MDP had a significant effect on promoting the development of nitrergic neurons. MDP had no significant effect on enteric neuronal precursor proliferation, apoptosis, or development into dopaminergic neurons. Development of serotonergic neurons was only significant between 0.01 µg/ml and 1 µg/ml, but not compared to the vehicle control (c). iE‐DAP had a significant effect on promoting the development of serotonergic and nitrergic neurons. iE‐DAP did not have a significant effect on enteric neuronal precursor proliferation, apoptosis, or development into dopaminergic neurons (d). *p ≤ .05. Values are presented as mean ± *SEM*

LPS treatment demonstrated an increase in the proliferation of HuC/D positive cells at 0.1 µg/ml, compared to vehicle control (*p* = .0405) and 0.001 µg/ml (*p* = .0242) (Figure 2a). A concentration‐dependent increase in development into serotonergic neurons (0.01 µg/ml, *p* = .0024; 0.1 µg/ml, *p* < .0001; 1 µg/ml, *p* = .0085) compared to the lowest does of LPS (0.001 µg/ml) was also observed; however, there was no significant difference compared to vehicle control (*p *> .05). There was a concentration‐dependent increase in development into nitrergic neurons (0.01 µg/ml, *p* = .0035; 0.1 µg/ml, *p* < .0001; 1 µg/ml, *p* = .0009) compared to the lowest concentrations of LPS (0.001 µg/ml), but not vehicle control (*p *> .05). A significant increase in nitrergic neurons between 0.1 µg/ml of LPS compared to vehicle control (*p* = .0198) was observed.

Flagellin demonstrated a concentration‐dependent increase in proliferation compared to vehicle control at 0.1 µg/ml (*p* = .0189) and 1 µg/ml (*p* = .0003) (Figure 2b). A concentration‐dependent increase in proliferation was also observed between 0.001 µg/ml and 0.1 µg/ml (*p* = .0497), 0.001 µg/ml and 1 µg/ml (*p* = .0003), and 0.01 µg/ml and 1 µg/ml (*p* = .0173). An increase in the proportion of serotonergic neurons was observed at 0.1 µg/ml (*p* = .0074) and 1 µg/ml (*p* = .0055), compared to vehicle control. There was a significant increase in the proportion of nitrergic neurons at 1 µg/ml of flagellin compared to vehicle control (*p* = .0132).

Cultures treated with MDP demonstrated a significant increase in the proportion of serotonergic neurons between 0.01 µg/ml and 1 µg/ml of MDP (*p* = .0178), but no significance was compared to vehicle control (Figure 2c). A significant increase in nitrergic neurons was found between vehicle control and 0.1 µg/ml of MDP (*p* = .0038), and vehicle control and 1 µg/ml of MDP (*p* = .0401). There was no significant difference in the proportion of proliferating cells at any concentration of MDP exposure.

Following iE‐DAP exposure, there was a significant increase in the proportion of serotonergic neurons between vehicle control and 10 µg/ml of iE‐DAP (*p* = .0233) (Figure 2d). This increase was also found between 1 µg/ml of iE‐DAP and 10 µg/ml of iE‐DAP (*p* = .0341). There was a significant increase in the proportion of nitrergic neurons between vehicle control and 0.1 µg/ml of iE‐DAP (*p* = .0151), and vehicle control and 1 µg/ml of iE‐DAP (*p* = .0153). There was no significant difference in the proportion of proliferation cells at any concentration of iE‐DAP tested.

No significant differences were found in apoptosis or the proportion of dopaminergic neurons at any concentration of bacterial components tested.

## 4 DISCUSSION

We tested the hypothesis that bacterial components directly interact with enteric neuronal precursors and influence the developmental programming of enteric neurons. We generated a model of enteric neuronal precursors in culture consistent with a perinatal period of development (Bergner et al., 2014). Subsequent treatment of the cultures with bacterial components provided evidence for direct interactions with developing enteric neurons. Specifically, the development of nitrergic neurons appears to be affected by all bacterial components tested in this study, including LPS, flagellin, and peptidoglycan derivatives, MDP, and iE‐DAP. Furthermore, flagellin and iE‐DAP appear to increase the proportion of serotonergic neurons. Finally, exposure to LPS and flagellin appear to stimulate proliferation in a population of enteric neuronal precursors.

Serotonergic neurons have been shown to be among the first phenotype of enteric neurons to emerge in the developing gut (Bergner et al., 2014; Pham et al., 1991). In contrast, dopaminergic neurons appear to consist of a heterogeneous population of both, early and late‐born neurons (Bergner et al., 2014). A population of transient catecholaminergic (TC) neurons was first shown to arise in the foregut at E9.5 (Baetge & Gershon, 1989; Teitelman et al., 1981), which is ultimately replaced by mature neurons by E14‐E15 (Baetge & Gershon, 1989). More recent evidence demonstrates the most abundance of dopaminergic neurons between E13.5‐E15.5 (Bergner et al., 2014; Li et al., 2004), followed by a second smaller peak around P0, with continued development until approximately P10 (Bergner et al., 2014). Similarly, the emergence of nitrergic neurons spans both, prenatal and postnatal timepoints, with the first subset of mature neurons emerging at E11.5 (Bergner et al., 2014; Hao et al., 2010), and completion of development at P10 (Bergner et al., 2014). When we compare actual percentages of enteric neurons from our subcultures to previous detailed work in whole‐mount preparations, we note that our nitrergic (10.00% ± 2.96%) and dopaminergic (0.180% ± 0.410%) populations in subculture 5 are consistent with the approximately 13.0% nitrergic neurons and < 1% dopaminergic neurons found at P0 (similar data at P0 not provided for serotonergic neurons) (Bergner et al., 2014). Given that the baseline characteristics of our cultures were suggestive of a perinatal timepoint, our findings suggest that populations of serotonergic and nitrergic neurons continue to be plastic and amenable to influences by intestinal bacteria past their previously established birthdate timelines.

We did not observe any significant effects of bacterial components on the development of dopaminergic neurons. This suggests that dopaminergic neurons may not be as responsive to microbial components as serotonergic and nitrergic neurons; instead, their development might be influenced by other endogenous factors. It has previously been shown that 5‐HT may affect the development of perinatal dopaminergic neurons (Fiorica‐Howells et al., 1998; Li et al., 2004), as mice lacking tryptophan hydroxylase 2 (TPH2) expression, an enzyme involved in serotonin synthesis, exhibited a reduction in dopaminergic populations (Li et al., 2011). Furthermore, 5‐HT dependent increases in the total numbers of enteric neurons in culture have also been shown, suggesting that the effects of 5‐HT on promoting enteric neuron survival and development are not restricted to dopaminergic neurons (Li et al., 2011). Indeed, 5‐HT may also have effects on neurons born later in the developmental period such as GABAergic, CGRP‐expressing, and even a subset of nitrergic neurons born after E15.5 (Li et al., 2011).

Enteric neuronal precursors that express both HuC/D and a marker of proliferation (pH3) comprise an intriguing population. We interpret this population of cells as being neuronal precursors that have yet to exit the cell cycle. Populations of early and late‐born neurons arise due to variations in the timing of cell cycle exit (Chalazonitis et al., 2008; Pham et al., 1991). Hu proteins have been shown to affect neuronal differentiation (Akamatsu et al., 2005; Ratti et al., 2006; Sakakibara & Okano, 1997), with the further suggestion that HuD might commence its expression in proliferative neuronal progenitor cells and then become a driver of cell cycle exit (Akamatsu et al., 2005). We hypothesize, therefore, that the cells expressing HuC/D and pH3 are committed neuronal precursors at the transition from final phases of mitosis and nearing cell cycle exit with development into specific neuronal phenotypes. As our current study suggests that this population of neuronal precursors can be influenced by external factors, and in particular by LPS and flagellin, further studies are needed. It would be interesting to determine, for example, if analogous to the ability of bone morphogenetic protein to regulate enteric phenotype diversity by promoting the exit of precursors from the cell cycle (Chalazonitis et al., 2008), bacterial components might influence enteric phenotype diversity by delaying cell cycle exit and thus the timing of neuronal birth dates. Indeed, previous work has suggested that different enteric subtypes might be specified by extrinsic factors acting during the time period of enteric precursor exit of the cell cycle (Bergner et al., 2014).

Finally, we demonstrated a lack of enteric neuronal precursor apoptosis across all bacterial component conditions and concentrations. These findings consist of the previously established concept that apoptosis is not an inherent characteristic of ENCDC maturation and ENS development, and that myenteric neurons are largely static until factors such as the onset of disease result in cell death (Kulkarni et al., 2017). A marked lack of apoptosis at a range of developmental stages from E12.5 to adult has been demonstrated using cleaved caspase‐3 staining in mouse myenteric plexus whole‐mount preparations from mice (Gianino et al., 2003). Using a different approach, a limited response in apoptosis to neurotrophin‐3 depletion on cultured ENCDCs has been demonstrated with no disturbances in the proportion of viable neurons using the TUNEL method (Chalazonitis et al., 2001).

While apoptosis is not a prominent process in the early development of the ENS, recent work suggests that apoptosis could play a role in the adult ENS. Kulkarni and colleagues (2017) showed that approximately a tenth of all myenteric neurons are tagged at all times for caspase‐3 cleavage and that roughly a third of these cells undergoes apoptosis within 7 days (Kulkarni et al., 2017), which is consistent with previous findings in other neuron types (Schreiber et al., 2007). Interestingly, apoptosis has also been detected in pre‐enteric vagal neural crest cells (NCCs) of an embryonic chick model, as they migrate toward the foregut and prior to foregut invasion (Wallace et al., 2009). Target cells were identified by immunohistochemistry with NCC marker HNK‐1 and either TUNEL or activated caspase‐3 to confirm apoptosis in NCCs that give rise to the ENS (Wallace et al., 2009). Taken together, these findings indicate that apoptosis is not a typical characteristic of enteric precursors during development after the invasion of the foregut; however, apoptosis may be a normal process in ENCDC precursors migrating from the vagal neural crest as well as adult myenteric neurons, as is typical of other nerves within the nervous system.

The findings presented here suggest that exposure of enteric neuronal precursors to bacterial components in perinatal development may directly influence the developmental programming of a subset of enteric neurons, particularly serotonergic and nitrergic neurons. We did not detect a significant influence on dopaminergic neurons; however, in accordance with previous research which demonstrated that serotonin may influence the proportions of dopaminergic neurons, it remains to be determined whether changes in chemical coding in early life could exert longer term effects. While our current work has focused on the development of enteric neurons, we recognize that enteric glia might also be similarly influenced by bacterial components (Kabouridis et al., 2015); this hypothesis could be an exciting area of future study. Overall, our work highlights the perinatal period as a critical timepoint during which the bacterial milieu can have direct effects on developing enteric neurons in early life, with the potential for longer term consequences, the cellular mechanisms of which remain to be elucidated.

## CONFLICT OF INTEREST

The authors wish to declare no conflicts of interest relevant to the conducted research.

## AUTHOR CONTRIBUTIONS

JP designed the project, conducted the experiments, analyzed the data, and produced the manuscript, JB conducted the experiments and analyzed the data, FM assisted with the immunocytochemistry data analyses, RB assisted with the project design and conducted the experiments, VA performed flow cytometry analyses, NP assisted with the preparation of the manuscript and statistical analyses, ER designed the project, obtained funding, provided essential tools for the experiments, and wrote the manuscript.

## References

[phy214611-bib-0001] Akamatsu, W. , Fujihara, H. , Mitsuhashi, T. , Yano, M. , Shibata, S. , Hayakawa, Y. , …, Okano, H. (2005). The RNA‐binding protein HuD regulates neuronal cell identity and maturation. Proceedings of the National Academy of Sciences of the United States of America, 102(12), 4625–4630. 10.1073/pnas.0407523102 15764704 PMC555491

[phy214611-bib-0002] Anitha, M. , Reichardt, F. , Tabatabavakili, S. , Nezami, B. G. , Chassaing, B. , Mwangi, S. , …, Srinivasan, S. (2016). Intestinal dysbiosis contributes to the delayed gastrointestinal transit in high‐fat diet fed mice. Cellular and Molecular Gastroenterology and Hepatology, 2(3), 328–339. 10.1016/j.jcmgh.2015.12.008 27446985 PMC4945127

[phy214611-bib-0003] Anitha, M. , Vijay‐Kumar, M. , Sitaraman, S. V. , Gewirtz, A. T. , & Srinivasen, S. (2012). Gut microbial products regulate murine gastrointestinal motility via toll‐like receptor 4 signaling. Gastroenterol, 143(4), 1006–1016. 10.1053/j.gastro.2012.06.034 PMC3458182 22732731

[phy214611-bib-0004] Arciszewski, M. B. , Sand, E. , & Ekblad, E. (2008). Vasoactive intestinal peptide rescues cultured rat myenteric neurons from lipopolysaccharide induced cell death. Regulatory Peptides, 146(1–3), 218–223. 10.1016/j.regpep.2007.09.021 17919746

[phy214611-bib-0005] Baetge, G. , & Gershon, M. D. (1989). Transient catecholaminergic (TC) cells in the vagus nerves and bowel of fetal mice: relationship to the development of enteric neurons. Developmental Biology, 132(1), 189–211. 10.1016/0012-1606(89)90217-0 2563710

[phy214611-bib-0006] Bergner, A. J. , Stamp, L. A. , Gonsalvez, D. G. , Allison, M. B. , Olson, D. P. , Myers, M. G. , …, Young, H. M. (2014). Birthdating of myenteric neuron subtypes in the small intestine of the mouse. The Journal of Comparative Neurology, 522(3), 514–527. 10.1002/cne.23423 23861145 PMC3877185

[phy214611-bib-0007] Binder, E. , Natarajan, D. , Cooper, J. , Kronfli, R. , Cananzi, M. , Delalande, J.‐M. , …, Thapar, N. (2015). Enteric neurospheres are not specific to neural crest cultures: Implications for neural stem cell therapies. PLoS One, 10(3), e0119467 10.1371/journal.pone.0119467 25799576 PMC4370605

[phy214611-bib-0008] Branchek, T. A. , & Gershon, M. D. (1989). Time course of expression of neuropeptide Y, calcitonin gene‐related peptide, and NADPH diaphorase activity in neurons of the developing murine bowel and the appearance of 5‐hydroxytryptamine in mucosal enterochromaffin cells. The Journal of Comparative Neurology, 285(2), 262–273. 10.1002/cne.902850208 2788179

[phy214611-bib-0009] Burgueño, J. F. , Barba, A. , Eyre, E. , Romero, C. , Neunlist, M. , & Fernández, E. (2016). TLR2 and TLR9 modulate enteric nervous system inflammatory responses to lipopolysaccharide. Journal of Neuroinflammation, 13(1), 1–15. 10.1186/s12974-016-0653-0 27538577 PMC4990868

[phy214611-bib-0010] Burns, A. J. , Goldstein, A. M. , Newgreen, D. F. , Stamp, L. , Schäfer, K.‐H. , Metzger, M. , … Vanden Berghe, P. (2016). White paper on guidelines concerning enteric nervous system stem cell therapy for enteric neuropathies. Developmental Biology, 417(2), 229–251. 10.1016/j.ydbio.2016.04.001 27059883 PMC5026875

[phy214611-bib-0011] Burns, A. J. , & Le Douarin, N. M. (1998). The sacral neural crest contributes neurons and glia to the post‐umbilical gut: Spatiotemporal analysis of the development of the enteric nervous system. Development, 125, 4335–4347. 9753687 10.1242/dev.125.21.4335

[phy214611-bib-0012] Chalazonitis, A. , Pham, T. D. , Li, Z. , Roman, D. , Guha, U. , Gomes, W. , …, Gershon, M. D. (2008). Bone morphogenetic protein regulation of enteric neuronal phenotypic diversity: Relationship to timing of cell cycle exit. The Journal of Comparative Neurology, 509(5), 474–492. 10.1002/cne.21770 18537141 PMC2592098

[phy214611-bib-0013] Chalazonitis, A. , Pham, T. D. , Rothman, T. P. , DiStefano, P. S. , Bothwell, M. , Blair‐Flynn, J. , …, Gershon, M. D. (2001). Neurotrophin‐3 is required for the survival–differentiation of subsets of developing enteric neurons. The Journal of Neuroscience, 21(15), 5620–5636. 10.1523/JNEUROSCI.21-15-05620.2001 11466433 PMC6762643

[phy214611-bib-0014] Collins, J. , Borojevic, R. , Verdu, E. F. , Huizinga, J. D. , & Ratcliffe, E. M. (2014). Intestinal microbiota influence the early postnatal development of the enteric nervous system. Neurogastroenterology and Motility, 26(1), 98–107. 10.1111/nmo.12236 24329946

[phy214611-bib-0015] Conner, P. J. , Focke, P. J. , Noden, D. M. , & Epstein, M. L. (2003). Appearance of neurons and glia with respect to the wavefront during colonization of the avian gut by neural crest cells. Developmental Dynamics, 226(1), 91–98. 10.1002/dvdy.10219 12508228

[phy214611-bib-0016] Coquenlorge, S. , Duchalais, E. , Chevalier, J. , Cossais, F. , Rolli‐Derkinderen, M. , & Neunlist, M. (2014). Modulation of lipopolysaccharide‐induced neuronal response by activation of the enteric nervous system. Journal of Neuroinflammation, 11, 202 10.1186/s12974-014-0202-7 25497784 PMC4279994

[phy214611-bib-0017] Crone, S. A. , Negro, A. , Trumpp, A. , Giovannini, M. , & Lee, K. F. (2003). Colonic epithelial expression of ErbB2 is required for postnatal maintenance of the enteric nervous system. Neuron, 37(1), 29–40. 10.1016/S0896-6273(02)01128-5 12526770

[phy214611-bib-0018] Fiorica‐Howells, E. , Maroteaux, L. , & Gershon, M. D. (1998). 5‐HT2B receptors are expressed by neuronal precursors in the enteric nervous system of fetal mice and promote neuronal differentiation. Annals of the New York Academy of Sciences, 15(861), 246. 10.1111/j.1749-6632.1998.tb10203.x 9928269

[phy214611-bib-0019] Gianino, S. , Grider, J. R. , Cresswell, J. , Enomoto, H. , & Heuckeroth, R. O. (2003). GDNF availability determines enteric neuron number by controlling precursor proliferation. Development, 130(10), 2187–2198. 10.1242/dev.00433 12668632

[phy214611-bib-0020] Hao, M. M. , Anderson, R. B. , Kobayashi, K. , Whitington, P. M. , & Young, H. M. (2009). The migratory behavior of immature enteric neurons. Developmental Neurobiology, 69(1), 22–35. 10.1002/dneu.20683 18985707

[phy214611-bib-0021] Hao, M. M. , Moore, R. E. , Roberts, R. R. , Nguyen, T. , Furness, J. B. , Anderson, R. B. , & Young, H. M. (2010). The role of neural activity in the migration and differentiation of enteric neuron precursors. Neurogastroenterology and Motility, 22(5), e127–e137. 10.1111/j.1365-2982.2009.01462.x 20082666

[phy214611-bib-0022] Hao, M. M. , & Young, H. M. (2009). Development of enteric neuron diversity. Journal of Cellular and Molecular Medicine, 13(7), 1193–1210. 10.1111/j.1582-4934.2009.00813.x 19538470 PMC4496134

[phy214611-bib-0023] Jiang, Y. , Liu, M. T. , & Gershon, M. D. (2003). Netrins and DCC in the guidance of migrating neural crest‐derived cells in the developing bowel and pancreas. Developmental Biology, 258(2), 364–384. 10.1016/S0012-1606(03)00136-2 12798294

[phy214611-bib-0024] Kabouridis, P. , Lasrado, R. , McCallum, S. , Chng, S. , Snippert, H. , Clevers, H. , …, Pachnis, V. (2015). Microbiota controls the homeostasis of glial cells in the gut lamina propria. Neuron, 85(2), 289–295. 10.1016/j.neuron.2014.12.037 25578362 PMC4306542

[phy214611-bib-0025] Kapur, R. P. , Yost, C. , & Palmiter, R. D. (1992). A transgenic model for studying development of the enteric nervous system in normal and aganglionic mice. Development, 116, 167–175. 1483385 10.1242/dev.116.Supplement.167

[phy214611-bib-0026] Kulkarni, S. , Micci, M.‐A. , Leser, J. , Shin, C. , Tang, S.‐C. , Fu, Y.‐Y. , …, Pasricha, P. J. (2017). Adult enteric nervous system in health is maintained by a dynamic balance between neuronal apoptosis and neurogenesis. Proceedings of the National Academy of Sciences of the United States of America, 114(18), E3709–E3718. 10.1073/pnas.1619406114 28420791 PMC5422809

[phy214611-bib-0027] Le Douarin, N. M. , & Teillet, M. A. (1973). The migration of neural crest cells to the wall of the digestive tract in avian embryo. Journal of Embryology and Experimental Morphology, 30(1), 31–48. 4729950

[phy214611-bib-0028] Li, Z. , Chalazonitis, A. , Huang, Y.‐Y. , Mann, J. J. , Margolis, K. G. , Yang, Q. M. , Kim, D. O. , …, Gershon, M. D. (2011). Essential roles of enteric neuronal serotonin in gastrointestinal motility and the development/survival of enteric dopaminergic neurons. Journal of Neuroscience, 31(24), 8998–9009. 10.1523/JNEUROSCI.6684-10.2011 21677183 PMC4442094

[phy214611-bib-0029] Li, Z. S. , Pham, T. D. , Tamir, H. , Chen, J. J. , & Gershon, M. D. (2004). Enteric dopaminergic neurons: Definition, developmental lineage, and effects of extrinsic denervation. Journal of Neuroscience, 24(6), 1330–1339. 10.1523/JNEUROSCI.3982-03.2004 14960604 PMC6730344

[phy214611-bib-0030] McKeown, S. J. , Chow, C. W. , & Young, H. M. (2001). Development of the submucous plexus in the large intestine of the mouse. Cell and Tissue Research, 303(2), 301–305. 10.1007/s004410000303 11291776

[phy214611-bib-0031] McVey Neufeld, K. A. , Perez‐Burgos, A. , Mao, Y. K. , Bienenstock, J. , & Kunze, W. A. (2015). The gut microbiome restores intrinsic and extrinsic nerve function in germ‐free mice accompanied by changes in calbindin. Neurogastroenterology and Motility, 27(5), 627–636. 10.1111/nmo.12534 25727007

[phy214611-bib-0032] Miyahara, K. , Kato, Y. , Koga, H. , Dizon, R. , Lane, G. J. , Suzuki, R. , Akazawa, C. , & Yamataka, A. (2011). Visualization of enteric neural crest cell migration in SOX10 transgenic mouse gut using time‐lapse fluorescence imaging. Journal of Pediatric Surgery, 46(12), 2305–2308. 10.1016/j.jpedsurg.2011.09.020 22152870

[phy214611-bib-0033] Neild, T. O. , Shen, K. Z. , & Surprenant, A. (1990). Vasodilation of arterioles by acetylcholine released from single neurones in the guinea‐pig submucosal plexus. Journal of Physiology, 420, 247–265. 2324984 10.1113/jphysiol.1990.sp017910 PMC1190047

[phy214611-bib-0034] Pham, T. D. , Gershon, M. D. , & Rothman, T. P. (1991). Time of origin of neurons in the murine enteric nervous system: Sequence in relation to phenotype. The Journal of Comparative Neurology, 314(4), 539–798. 10.1002/cne.903140411 1816276

[phy214611-bib-0035] Ratti, A. , Fallini, C. , Cova, L. , Fantozzi, R. , Calzarossa, C. , Zennaro, E. (2006). A role for the ELAV RNA‐binding proteins in neural stem cells: Stabilization of Msi1 mRNA. Journal of Cell Science, 119, 1442–1452. 10.1242/jcs.02852 16554442

[phy214611-bib-0036] Sakakibara, S. , & Okano, H. (1997). Expression of neural RNA‐binding proteins in the postnatal CNS: Implications of their roles in neuronal and glial cell development. Journal of Neuroscience, 17(21), 8300–8312. 10.1523/JNEUROSCI.17-21-08300.1997 9334405 PMC6573750

[phy214611-bib-0037] Sang, Q. , & Young, H. M. (1996). Chemical coding of neurons in the myenteric plexus and external muscle of the small and large intestine of the mouse. Cell and Tissue Research, 284(1), 39–53. 10.1007/s004410050565 8601295

[phy214611-bib-0038] Schreiber, K. L. , Price, L. D. , & Brown, D. R. (2007). Evidence for neuromodulation of enteropathogen invasion in the intestinal mucosa. Journal of Neuroimmune Pharmacology: The Official Journal of the Society on NeuroImmune Pharmacology, 2(4), 329–337. 10.1007/s11481-007-9087-x 18040851

[phy214611-bib-0039] Teitelman, G. , Gershon, M. D. , Rothman, T. P. , Joh, T. H. , & Reis, D. J. (1981). Proliferation and distribution of cells that transiently express a catecholaminergic phenotype during development in mice and rats. Developmental Biology, 86(2), 348–355. 10.1016/0012-1606(81)90192-5 6116632

[phy214611-bib-0040] Wallace, A. S. , Barlow, A. J. , Navaratne, L. , Delalande, J. M. , Tauszig‐Delamasure, S. , Corset, V. , …, Burns, A. J. (2009). Inhibition of cell death results in hyperganglionosis: Implications for enteric nervous system development. Neurogastroenterology and Motility, 21(7), 768 10.1111/j.1365-2982.2009.01309.x 19400926

[phy214611-bib-0041] Yntema, C. L. , & Hammond, W. S. (1954). The origin of intrinsic ganglia of trunk viscera from vagal neural crest in the chick embryo. The Journal of Comparative Neurology, 101(2), 515–541. 10.1002/cne.901010212 13221667

[phy214611-bib-0042] Young, H. M. , Ciampoli, D. , Hsuan, J. , & Canty, A. J. (1999). Expression of Ret‐, p75NTR‐, Phox2a‐, Phox2b‐, and tyrosine hydroxylase‐immunoreactivity by undifferentiated neural crest‐derived cells and different classes of enteric neurons in the embryonic mouse gut. Developmental Dynamics, 216(2), 137–152. 10.1002/(SICI)1097-0177(199910)216:2<137:AID-DVDY5>3.0.CO;2-6 10536054

[phy214611-bib-0043] Young, H. M. , Newgreen, D. , & Burns, A. J. (2006). The Development of the enteric nervous system in relation to Hirschsprung’s disease In Ferretti P., Copp A., Cheryll T., & Moore G. (Eds.), The Development of the enteric nervous system in relation to Hirschsprung’s disease (pp. 263–300). Chichester, West Sussex PO19 8SQ, England: John Wiley & Sons.

